# Factors That Affect Oral Care Outcomes for Institutionalized Elderly

**DOI:** 10.1155/2018/2478408

**Published:** 2018-12-10

**Authors:** Yoshiaki Nomura, Noriko Takei, Takanori Ishii, Koji Takada, Yasuharu Amitani, Hitomi Koganezawa, Shizuko Fukuhara, Keita Asai, Ryuji Uozumi, Kazuhisa Bessho

**Affiliations:** ^1^Department of Translational Research, Tsurumi University School of Dental Medicine, 2-1-3 Tsurumi, Tsurumi-ku, Yokohama 230-8501, Japan; ^2^The Lion Foundation for Dental Health, 3-7 Honjo 1-Chome, Sumida-ku 130-8644, Japan; ^3^Department of Mathematics, Tsurumi University School of Dental Medicine, 2-1-3 Tsurumi, Tsurumi-ku, Yokohama 230-8501, Japan; ^4^Medical Corporation Hakuoukai Koganezawa Dental Clinic, 5-6 Ooishihigashi 4-Chome, Otsu-Shi Siga 520-2264, Japan; ^5^Department of Oral and Maxillofacial Surgery, Graduate School of Medicine, Kyoto University, 54 Kawahara-Cho, Sakyo-Ku, Kyoto 606-8507, Japan; ^6^Department of Biomedical Statistics and Bioinformatics, Graduate School of Medicine, Kyoto University, 54 Kawahara-Cho, Sakyo-Ku, Kyoto 606-8507, Japan

## Abstract

The aim of this study was to evaluate the effect of an oral care intervention program on the incidence of pneumonia and fever as a surrogate endpoint. In addition, we tried to determine the oral care risk factors for the incidence of fever. We provided an oral care program for the elderly at one private nursing home in July 2013. The maximum capacity of the nursing home was 60 residents. The body temperatures of all residents were measured twice a day and were summarized as the incidence of fever over a one-month period, which was used as the dependent variable. The residents' life conditions, number of teeth, and prescribed diet were used as independent variables. The factors that affected the incidence of fever were the number of remaining teeth, a prescribed diet of sliced food, the meal care level, and the oral *Candida* levels. These risk factors affected the incidence of fever independently or interactively with oral care. Some risk factors for the incidence of fever were enhanced by the oral care program. It is important to evaluate and control these factors before the implementation of an oral care program.

## 1. Introduction

Aspiration pneumonia is a major etiology of morbidity and mortality in both independent-living and institutionalized adults over 60 years of age [1, 2]. Residents in long-term care facilities are at a particularly high risk [3, 4]. Aspiration pneumonia ranked first in mortality and second in morbidity among nosocomial infections [5, 6]. Aspiration pneumonia refers to lower respiratory infection caused by the inhalation of oropharyngeal secretions colonized by pathogenic bacteria [1]. A major precursor of pathogenic secretions is dental plaque; the elimination of dental plaque reduces bacterial colonization in pathogenic secretions. An epidemiological study showed that subjects with the maximum Oral Hygiene Index (OHI) score were 4.5 times more likely to have a chronic respiratory disease than those with an OHI of 0 [7]. Therefore, oral care is widely regarded as a crucial, common, and cost-effective method for preventing aspiration pneumonia [5, 6, 8].

In the initial stages of an aging or super-aging society, oral care for the institutionalized elderly is a niche field for the prevention of diseases, especially pneumonia. Innovative reports have shown that a combination of oral care provided by dental professionals and daily oral care provided by nurses or caregivers was effective for preventing fever, new-onset pneumonia, and pneumonia-related death [9, 10].

Several strategies for preventing aspiration pneumonia have been proposed, and putative risk factors related to oral care have been investigated [11–16]. In addition, medical conditions other than oral health status have been investigated [11, 17–20], and these reports indicated that critical factors other than oral health status may independently increase the risk of fever and pneumonia.

An innovative report [9, 10] showed that oral care intervention plays an important role in preventing aspiration pneumonia and subsequent hospitalization and death, and several reports have reinforced this evidence [21]. However, rebuttals of this evidence have also been published, including findings that subjects receiving oral care had a higher risk of death from pneumonia [20] and that no relationship existed between acute respiratory diseases and oral health status [7]. Additionally, the results of a randomized controlled trial showed that compared with the usual care, oral care interventions provided by nondental professionals did not significantly reduce the incidence of pneumonia in nursing home residents [22]. Finally, a recent systematic review and meta-analysis of randomized controlled trials concluded that while oral care interventions provided by dental personnel reduced mortality from pneumonia, oral care interventions provided by nursing personnel did not result in a statistically significant difference [23].

In the case of daily oral care, interprofessional collaboration can offer solutions. However, although current critical care nursing manuals [24–28] and protocols issued by The American Association for Critical Care Nurses are available [29], oral care is not universally practiced by nurses [30–32].

We planned and carried out a preliminary follow-up study to evaluate the effects of an oral care program, with the incidence of pneumonia and fever as the surrogate endpoint. However, negative results were obtained. In this study, we used statistical modeling to analyze the reason for this result and tried to determine the oral care-related risk factors for fever.

## 2. Materials and Methods

### 2.1. Study Design and Setting

An intervention and follow-up study was conducted to evaluate the effects of an oral care program on the incidence of fever in institutionalized elderly adults. We applied an oral care program for the elderly in July 2013 at one private nursing home located in a suburban area of Osaka, Japan. The maximum capacity of this facility was 60 residents.

### 2.2. Participants

In July 2013, 60 elderly adults resided in the facility. We recommended an oral care program for all the residents. Seven residents who could not undergo the oral examination due to being hospitalized, dementia, another condition that prevented them from giving fully informed consent, or complex medical problems were excluded from the analysis. At the start of the oral care program, informed consent could not be obtained from eight subjects; however, they all agreed to participate in the oral care program within six months after the start of the program.

Because of the relatively high fee for this facility compared with public facilities, some of the residents moved to public institutions. Therefore, the residents' duration of stay varied in part because of the residents' financial backgrounds. Summary statistics for duration of stay from July 2013 to June 2016 were as follows: mean: 25.2 months, range: 3–36 months, and median: 25.0 months, including censored cases from June 2016. Within one year, from July 2013 to June 2014, 24 residents left the facility and 26 moved in. A total of 81 subjects were observed, and the oral care program was accepted by 72 subjects. These 72 subjects (15 men, 57 women, mean age: 85.4+/−8.5 years) were analyzed for the duration or until the incidence of fever. Fifty-three subjects (10 men, 43 women, mean age: 84.3+/−8.4 years) who could be followed for more than 12 months were analyzed; among them, twenty-seven subjects (5 men, 22 women, mean age: 84.2+/−7.2 years) with available data for one year before the start of the oral care program were analyzed to evaluate the effect of the oral care program and associated factors. The duration of stay for these subjects is illustrated in Figure S1.

### 2.3. Diagnosis of Pneumonia and Fever

The nurses employed at the nursing homes routinely took the body temperatures of all residents twice every day, at 7 : 00 and 15 : 00. Residents with a body temperature of 37.8°C or above were considered to be feverish [33]. Subjects with suspected pneumonia were sent to hospitals. At the hospital, pneumonia was diagnosed based on the following standard criteria: fever (body temperature ≥37.8°C), high CRP level, and an infiltration shadow on chest X-rays and/or computed tomography [34].

### 2.4. Physical Conditions, Diet and Food Prescriptions, Oral Function, Independence of Oral Care

The care level and life conditions (including food intake and meal styles) were obtained from the residential records. The incidence of fever and hospitalization for pneumonia was also obtained from the daily residential medical records. The diet- and food-related factors investigated in this study were meal care (independent, needs attention during meals, and dependent), prescribed diet (ordinary foods, sliced foods, pureed foods, and liquid foods), and the presence or absence of arousal during the meal.

In this study, participants who needed attention during meals were defined as those who had dysphagia but took food independently. The consciousness level was evaluated as the presence or absence of arousal during the meal.

Independence of oral care was evaluated in terms of toothbrushing (independent, needs attention during toothbrushing, and dependent). Participants who needed attention during toothbrushing were defined as those who had dysphagia but could brush their teeth independently.

Swallowing function in subjects with dysphagia was evaluated by swallowing 75 g of specialized gel (Engeread Apple Gel, Otsuka, Tokushima, Japan). The dentist determined the number of swallows and the presence of residue in the oral cavity. The diagnostic categories were without residue, with residue, and inability to swallow.

### 2.5. Oral Examination

Oral examinations were performed by one dentist (K.K.). The number of remaining teeth was recorded. As many patients had remaining roots, the number of teeth with intact crowns was counted and used as the number of functional teeth in the following analyses.

### 2.6. *Candida* Sampling and Culture

*Candida* samples were obtained from tongue by swabbing the central area of the tongue surface 10 times with sterile cotton sticks. The samples were smeared onto BBL CHROMagar™ *Candida* medium (BD Biosciences10) and incubated at room temperature for 48 hours.

### 2.7. Oral Care Program Management

For the elderly, nurses, and caregivers, toothbrushing instruction that aimed to improve efficiency and reduce insufficient brushing was provided. Independent and partially dependent denture wearers typically washed their mouths with water, while dependent denture wearers' oral cavities were wiped with a sponge brush. For these subjects, a specialized brush for the oral mucosa (Dent ELAC 510 LION, Tokyo, Japan) was introduced. For dentures, which were typically washed with water, the use of a denture cleaner (Dent ELAC LION, Tokyo, Japan) and a denture brush (Dent ELAC 710M LION, Tokyo, Japan) was introduced.

### 2.8. Sample Size Estimation

Based on the data from a previous report [10], the sample sizes needed to detect a difference in the incidence of fever and pneumonia as a result of the oral health program were 122 and 271 per group for the fever and pneumonia, respectively. However, for ethical reasons, it was not possible to collect data at one facility without providing the oral care program. Therefore, we consider this a preliminary study.

### 2.9. Statistical Methods

The incidence of fever as a major outcome was used to evaluate the oral care program. Because with or without the oral care program, data status was nested within each subject, and mixed effects modeling [35] was applied for the 27 subjects for whom data from one year before and one year after the start of the oral care program were available. As the distributions of the incidence of fever and pneumonia were skewed, Poisson distribution was used for the probability distribution. Applying a constructed mixed effect model, the incidence of fever was simulated by oral care and the number of functional teeth. For the fifty-five subjects who accepted oral care and could be followed for at least one year, their characteristics at baseline and after 6 months and 12 months were compared and *P* values were calculated with Friedman tests. The risk factors for the incidence of fever were analyzed with a generalized linear model, and the Poisson distribution was used for the probability distribution. For the seventy-two subjects who accepted oral care, the duration until the incidence of fever was analyzed by Cox's proportional hazard model and survival curves were illustrated using a life table. These analyses were performed using SPSS Statistics ver. 24.0 and SPSS Modeler ver. 18.0 (IBM Japan, Tokyo, Japan). The models, including the SPSS syntaxes, are specified in Supplementary File/Figure S1.

## 3. Results

Time series plots of the incidence of fever and pneumonia-related hospitalization are presented in Figure S2. The incidences of both fever and pneumonia-related hospitalization increased after the start of oral care, and the development of fever and pneumonia-related hospitalization appeared to be parallel.

**Figure 1:**
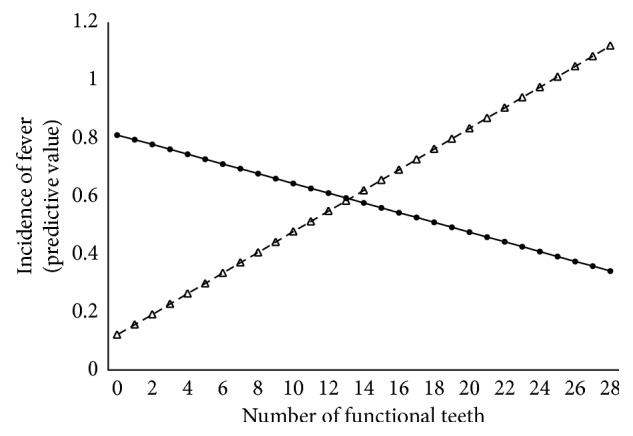
Predictive value of the incidence of fever within one year with or without oral care. The solid line indicates subjects who received oral care, and the dotted line indicates subjects who did not receive oral care. For the subjects who received the oral care program, the predictive value for the incidence of fever decreased with the increase in the number of functional teeth. In contrast, for the subject who did not receive oral care, the predictive values for the incidence of fever increased with the increase of number of functional teeth.

We analyzed the effect of the oral care program on the twenty-seven subjects who had been institutionalized for more than one year before and after start of the oral care program. The characteristics of the subjects at the start of the oral care program are presented in Table S1. The distributions of the incidence of fever and pneumonia-related hospitalization over one year are shown in Figure S3. The distributions were skewed. As incidences were nested within each subject, we applied the mixed effects model to evaluate the effects of the oral care program and its associated factors. Poisson distributions were used for the probability distributions.  Table 1 shows the results for the effect of the oral care program, the number of functional teeth, and their interactions on fever and hospitalization for pneumonia. The oral care program had a statistically significant effect on fever; its coefficient was positive, as was the coefficient of the number of functional teeth. In contrast, the coefficient for the interaction of oral care and the number of functional teeth was negative. However, the oral care program did not have a significant effect on hospitalization for pneumonia. Therefore, we simulated the incidence of fever using Model 1 (A), presented in Table 1. The results are illustrated in Figure 1. For the subjects receiving the oral care program, the predictive values of the incidence of fever decreased with the increased number of functional teeth. In contrast, for the subjects who did not receive care, the predictive values for the incidence of fever increased with the increased number of functional teeth. The effects of other oral health-related factors are presented in Table S2. Denture use was not a statistically significant factor. The need for attention and dependence for toothbrushing was statistically significant. The inability to swallow was a statistically significant factor.

We then analyzed the factors that affect the incidence of fever based on the subjects' characteristics at the start of the oral care. Factors related to dietary intake or meals were included in Model 1 (A) separately. The results are shown in Table 2 as Model 2. As Model 2 (A) shows, there was a higher risk of fever among the subjects who needed attention during meals than among those who did not need assistance. According to Model 2 (B), there was a higher risk among the subjects who were prescribed sliced food than among those who were prescribed pureed food. As the model included the oral care program and the number of functional teeth, the effects of these factors on fever were independent of the oral care program and the number of functional teeth. Subsequently, we evaluated the interaction effect between these factors and the intervention or the number of functional teeth on fever. The results are presented in Table S3. The interactions between the need for attention at meal times, participation in the oral care program, nonarousal during the meal, and not undergoing the oral care program showed statistically significant risks for fever. Interactions between the food prescription and the oral care program were not associated with a statistically significant risk of fever. In contrast, interactions between the number of functional teeth and ordinary and sliced food prescriptions were associated with a risk of fever. The coefficient for sliced food prescriptions was higher than that for ordinary food.

Our investigation showed that oral levels of *Candida* can be an indicator for the risk of fever. The data of oral levels of *Candida* were dichotomized (cutoff point: 750 cfu). The procedure for establishing the cutoff point is described in Supplementary File/Figure S2. As Table 3 shows, coefficient for *Candida* (+) was statistically significant. The effects of the interactions between oral levels of *Candida* and the oral care program or the number of functional teeth on fever are shown in Table S4. Statistically significant coefficients were obtained for interaction between oral care (+) and *Candida* (+). The results indicated that the risk of fever may increase when oral care is provided for subjects with higher levels of *Candida*. In addition, interactions between the number of functional teeth and *Candida* had a statistically significant coefficient. The coefficient for *Candid*a levels ≥750 cfu was higher than that for levels <750 cfu.

For fever, dietary and meal prescription and oral levels of *Candida* were risk factors independent of oral care. The interaction effects for the oral care program and these factors were statistically significant. However, it was necessary to evaluate whether these factors could predict the risk of fever in subjects receiving oral care. We analyzed a total of 53 subjects who were institutionalized and had been receiving oral care for 12 months, including the 27 subjects analyzed above. The characteristics of the subjects at baseline, after 6 months, and after 12 months are described in Table S1. The incidences of fever, hospitalization, and hospitalization for pneumonia are shown in Figure S4. Multivariate adjusted generalized linear model analysis showed that the number of functional teeth, the need for attention at meal times, a sliced food prescription, and high levels of oral *Candida* (750 cfu≤) were risk factors for the incidence of fever in subjects participating in the oral care program (Table 4).

Finally, Cox's proportional hazard model was applied for the duration from the start of the oral care program until the incidence of fever. The hazard ratios of the subjects who were prescribed sliced food and those who did not show arousal during the meals were statistically significant and high. The hazard ratio of the subjects who needed attention during meals was relatively high; however, it was not statistically significant (Table 5). The survival curves for food prescription and meal care are illustrated in Figure S5.

## 4. Discussion

Oral health contributes general health conditions. It plays an especially important role in the prevention of pneumonia among institutionalized elderly adults. However, the provision of oral care to improve oral health is not always effective for preventing pneumonia. A previous systematic review indicated that the effect of oral care largely depends on the strategy and methodology of oral care used [23]. In this study, we determined confounding factors that have an effect on the incidence of fever as a surrogate endpoint of pneumonia by oral care. To prevent aggravation, it may be useful to take these confounding factors into account when planning an oral care program.

As Table 1 shows, the oral care program itself increased the risk of fever. However, the risk was decreased in accordance with the increased number of functional teeth. Previous reports have shown that the number of remaining teeth was a statistically significant risk factor for pneumonia [36], and the risk increased if the remaining teeth had decay [37]. As Table 1 (A) and Figure 1 show, our results demonstrated that the risk of fever increased in accordance with the increase in the number of functional teeth among the subjects who did not receive the oral care program. In contrast, the risk of fever decreased as the number of functional teeth increased. These results indicate that oral care intervention may be effective for dentate subjects. The intercept of the line in Figure 1 denotes edentulous subjects. For edentulous subjects and subjects with fewer than 13 remaining functional teeth, the oral health program was associated with a risk of fever. This result suggests that there may be confounding risk factors that enhance the risk of fever by interacting with the oral health program, especially for edentulous subjects. It is important to clarify any confounding issues.

Dysphagia is a major risk factor for aspiration pneumonia. Meal or food type can be risk factors for dysphagia and subsequent aspiration pneumonia, with their surrogate endpoint of fever. According to Models 2 (A), (B), and (C), the need for attention during the meal because of dysphagia, a sliced food prescription, and the absence of arousal during the meal were risk factors for fever. These three factors were independent risk factors for fever because including them in the model simultaneously with the oral care program would require the adjustment of the oral care program. The coefficients of these factors were higher than those of the oral care program and the number of functional teeth. A previous report showed that dependence for feeding [20] and needing help during eating [38] were risk factors for pneumonia. For the interactions need attention during meals and oral care, both with and without oral care program were statistically significant (Model S1 (A)). Need attention during meals was also statistically significant in the subjects under the oral care program (Model 4). In contrast, need assistance during the meal was not statistically significant in all the models presented in this study. This may be because dedicated attentions may be paid for dependent elderlies, and latent dysphagia may be often occurred for the elderlies need attention during the meals.

Food or meal style and ingredient of meal are different between the countries and traditions. Simple comparison of the research is difficult. Randomized control trials were conducted to evaluate the effect of food style for the incidence of pneumonia [38, 39]. However, clear information is still not available. In this study, interaction of prescription of sliced food and oral care program were not significant. In contrast, interaction of prescription of sliced food and number of functional teeth were significant (Model S2 (B)). The prescription of sliced food was a significant risk factor for the subjects under the oral care program (Models 4 and 5). The results indicate that the food prescription may act as a risk factor for the fever independently of the oral care.

Consciousness disorder is a risk factor of pneumonia. The coefficients of arousal during the meal were statistically significant, and the value of them were all higher than other two factors in all the models presented in this study. The interaction of without arousal and without oral care was statistically significant (Model S3 (C)). The result indicated that the oral care program may reduce the risk of fever efficiently for these subjects. However, as shown in Models 5 and 6, no arousal during the meal acts as a prominent risk factor for the fever for the subjects even under oral care program. As described above, these factors concerning the meal and food play an important role for the fever independent of the oral care. The incidence of fever, pneumonia, and subsequent death by pneumonia may be affected by these factors. The subjects with these characteristics may have large effect on the result of oral care program for the pneumonia.

Nursing home residents tended to have a high degree of dental plaque or denture plaque, which is a major cause of respiratory pathogen colonization [40]. The etiologic agents of nosocomial pneumonia are mainly Gram-negative bacilli and staphylococci. Routine analysis of these bacterial infections is difficult because of the technical difficulties associated with anaerobic culture and the possibility of contamination by anaerobic oral flora during sampling [41]. It was reported that, in addition to these Gram-negative bacilli and staphylococci, *Candida* rates were higher in nursing home residents than in the healthy elderly [42]. Professional oral health care provided by a dental hygienist effectively reduced the presence of *Candida albicans* [43]. Proper attention to oral hygiene, including brushing and rinsing the mouth, could reduce the susceptibility to infection by opportunistic pathogens, including *Candida* [44]. Oral levels of *Candida* may not be a direct cause of aspiration pneumonia; however, oral levels of *Candida* can be an indicator of oral hygiene concerns [45].

As Model S4 shows (B), for the subjects with higher levels of *Candida*, the oral care program and an increase in the number of functional teeth increased the risk of fever. The oral care program applied in this study did not include professional care by the dental hygienist or mouth rinses with antimicrobial or anti-antifungal agents. The result indicated that, for subjects with poor oral health, presumed based on a high level of *Candida*, the introduction of a self-administered oral care program may increase the risk of fever and subsequent pneumonia. One reason for this phenomenon may be that accumulated dental plaque containing pathogenic bacteria may be dispersed by inappropriate oral care. For these subjects, it may be necessary to provide antibiotics or care from dental professionals before introducing the oral care program.

Given the limited human resources of the dental professions, professional oral care cannot be provided for all of the elderly people living in nursing homes. This study showed the results of an oral care program for reducing the incidence of fever without professional care. We determined candidate risk factors that act independently or interact with oral care on the incidence of fever. However, the sample size was not large enough to construct more complex statistical models and evaluate the incidence of pneumonia. Larger samples are necessary to confirm the results obtained in this study.

In conclusion, some risk factors may be increased by the introduction of an oral care program. It may be important to evaluate and control these factors before the implementation of an oral care program.

## Figures and Tables

**Table 1:** Effect of oral care and number of functional teeth and their interaction on the incidence of fever and hospitalization for pneumonia.

		Model 1 (A)				Model 1 (B)			
		Fever				Hospitalization for pneumonia			
		Coefficient	95% CI		*P* value	Coefficient	95% CI		*P* value
			Lower	Upper			Lower	Upper	
*Intercept*		0.121	−0.140	0.382	0.355	0.173	−0.073	0.420	0.164
*Oral care*	−	Reference				Reference			
	+	0.691	0.110	1.272	0.021	0.298	−0.168	0.763	0.205
*Number of functional teeth*		0.036	0.004	0.067	0.029	−0.002	−0.02	0.015	0.774
*Interaction with number of functional teeth*									
*Oral care*	−	Reference				Reference			
	+	−0.052	−0.100	−0.005	0.030	−0.007	−0.039	0.024	0.636

(A): meal care, (B): food prescription. Data were analyzed using mixed effect modeling. The coefficients of oral care and number of functional teeth were positive. However, the coefficients of the interaction between oral care and number of functional teeth were negative. All coefficients for hospitalization for pneumonia were not statistically significant.

**Table 2:** Effect of dietary or meal factors on fever, adjusted by oral care and number of functional teeth.

		Model 2 (A)				Model 2 (B)				Model 2 (C)			
		Coefficient	95% CI		*P* value	Coefficient	95% CI		*P* value	Coefficient	95% CI		*P* value
			Lower	Upper			Lower	Upper			Lower	Upper	
*Intercept*		−0.063	−0.512	0.387	0.780	0.186	−0.168	0.541	0.296	−0.774	−1.71	0.163	0.103
*Oral care*	−	Reference				Reference				Reference			
	+	0.814	0.104	1.525	0.026	0.675	0.131	1.218	0.016	0.774	−0.163	1.712	0.102
*Number of functional teeth*		0.035	−0.009	0.078	0.118	0.023	−0.006	0.051	0.123	0.093	0.019	0.168	0.015	
*Interaction with number of functional teeth*													
*Oral care*	−	Reference				Reference				Reference			
	+	−0.061	−0.117	−0.005	0.033	−0.049	−0.089	−0.01	0.015	−0.087	−0.162	−0.011	0.025
*Meal care*													
Independent		Reference			
Needs attention		1.544	0.547	2.540	0.003									
Dependent		−0.342	−f0.837	0.153	0.170									
*Food prescription*													
Ordinary						Reference							
Sliced						1.043	0.003	2.084	0.049				
Pureed						−0.127	−0.457	0.203	0.442				
*Arousal during the meal*													
Arousal (+)										Reference			
Arousal (−)										1.762	0.658	2.867	0.003

(A): meal care, (B): food prescription, (C): arousal during the meal. The coefficients of subjects who needed attention during the meal, who were prescribed sliced food, and who were unaroused during meals were positive and statistically significant.

**Table 3:** Effect of oral levels of *Candida* on fever, adjusted by oral care and number of functional teeth.

		Model 3			
		Coefficient	95% CI		*P* value
			Lower	Upper	
*Intercept*		−0.125	−0.499	0.249	0.504
*Oral care*	−	Reference			
	+	0.640	0.047	1.233	0.035
*Number of functional teeth*		0.041	0.001	0.081	0.045
*Interaction with number of functional teeth*					
*Oral care*	−	Reference			
	+	−0.049	−0.100	0.002	0.059
*Oral levels of Candida*					
<750 cfu		Reference			
≥750 cfu		0.599	0.121	1.077	0.015

The cutoff point was set by describing ROC curves. Oral levels of *Candida* higher than 750 cfu were a risk factor for fever.

**Table 4:** Risk factors for fever among the subjects receiving the oral care program.

	Model 4			
	Coefficient	95% CI		*P* value
		Lower	Upper	
*Intercept*	−3.205	−4.850	−1.559	0.000
*Number of functional teeth*	0.084	0.013	0.154	0.020
*Meal care*				
Independent	Reference			
Needs attention	1.076	0.061	2.091	0.038
Needs assistance	−1.970	−4.004	0.064	0.058
*Food prescription*				
Ordinary	Reference			
Sliced	1.491	0.249	2.734	0.019
Pureed	1.063	−0.280	2.406	0.121
Liquid	−23.185	—	—	—
*Arousal during the meal*				
Arousal (+)	Reference			
Arousal (−)	1.770	0.537	3.004	0.005
*Candida*				
<750 cfu	Reference			
≥750 cfu	1.281	0.018	2.544	0.047

Fifty-three subjects who received oral care and could be followed for more than one year were analyzed by multilevel generalized linear regression. Statistically significant factors were almost the same as those of previous models.

**Table 5:** Risk factors associated with duration until the incidence of fever after starting the oral care program.

	Model 5			
	Hazard ratio	95% CI		*P* value
		Lower	Upper	
*Number of functional teeth*	0.997	0.940	1.056	0.909
*Meal care*				
Independent	Reference			0.353
Need attention	2.008	0.625	6.446	0.242
Need assistance	0.699	0.171	2.865	0.619
*Food prescription*				
Ordinal	Reference			0.113
Sliced	3.795	1.082	13.305	0.037
Pureed	1.958	0.616	6.226	0.255
*Arousal during the meal*				
Arousal (+)	Reference			
Arousal (−)	4.219	1.096	16.243	0.036
*Candida*				
≤750 cfu	Reference			0.353
≥750 cfu	0.846	0.298	2.408	0.755

The model was constructed by Cox's Proportional hazard model. No arousal during the meal (conscious level) and having sliced food were risk factors.

## Data Availability

Data are not published to protect the identity of our participants; however, they can be provided upon request by contacting the corresponding author.

## Supplementary Materials

Supplementary Materials S1: file model specification; S2: file cutoff point of oral levels of *Candida* for fever. S1 Table: characteristics of the subjects analyzed in this study at the start of oral care. S2 Table: effects of oral care independence on fever according to participation in the oral care program and number of functional teeth. S3 Table: effects of the interaction of dietary or meal factors with the oral care program and the number of functional teeth on the incidence of fever. S4 Table: effects of the interactions between *Candida* and oral care and *Candida* and the number of functional teeth on fever. Figure S1: the observational periods of the subjects who participated in this study. Figure S2: time series plots of the incidence of fever and hospitalization for pneumonia. Figure S3: distribution of the incidences of fever, hospitalization, and pneumonia-related hospitalization in patients who did and did not receive the oral care program. Figure S4: distribution of the incidence of fever and pneumonia-related hospitalization over the one-year follow-up period among the subjects who underwent the oral care program. Figure S5: probability of remaining fever-free among the subjects receiving the oral care program.

## References

[1] Granton J. T., Grossman R. F. (1993). Community-acquired pneumonia in the elderly patient. Clinical features, epidemiology, and treatment. *Clinics in Chest Medicine*.

[2] Niederman M. S. (1993). Nosocomial pneumonia in the elderly patient. Chronic care facility and hospital considerations. *Clinics in Chest Medicine*.

[3] Marik P. E. (2001). Aspiration pneumonitis and aspiration pneumonia. *New England Journal of Medicine*.

[4] Dogget D. L., Tappe K. A., Mitchell M. D. (2001). Prevention of pneumonia in elderly stroke patients by systematic diagnosis and treatment of dysphagia: an evidence-based comprehensive analysis of the literature. *Dysphagia*.

[5] Yoon M. N., Steele C. M. (2012). Health care professionals’ perspectives on oral care for long-term care residents: nursing staff, speech-language pathologists and dental hygienists. *Gerodontology*.

[6] Ueda K. (2011). Preventing aspiration pneumonia by oral healthcare. *Japan Medical Association Journal*.

[7] Scannapieco F. A., Papandonatos G. D., Dunford R. G. (1998). Associations between oral conditions and respiratory disease in a national sample survey population. *Annals of Periodontology*.

[8] Franceschini T. (2009). Oral care. *Adv Speech Hearing*.

[9] Yoneyama T., Yoshida M., Matsui T., Sasaki H. (1999). Oral care and pneumonia. *The Lancet*.

[10] Oral Care Working Group (2002). Oral care reduces pneumonia in older patients in nursing homes. *Journal of the American Geriatrics Society*.

[11] Loeb M. B., Becker M., Eady A., Walker-Dilks C. (2003). Interventions to prevent aspiration pneumonia in older adults: a systematic review. *Journal of the American Geriatrics Society*.

[12] Bansal M., Khatri M., Taneja V. (2013). Potential role of periodontal infection in respiratory diseases: a review. *Journal of Medicine and Life*.

[13] Garcia R. (2005). A review of the possible role of oral and dental colonization on the occurrence of health care-associated pneumonia: underappreciated risk and a call for interventions. *American Journal of Infection Control*.

[14] Hoshijima H., Kuratani N., Takeuchi R. (2013). Effects of oral hygiene using chlorhexidine on preventing ventilator-associated pneumonia in critical-care settings: a meta-analysis of randomized controlled trials. *Journal of Dental Sciences*.

[15] Mojon P. (2002). Oral health and respiratory infection. *Journal of the Canadian Dental Association*.

[16] Quagliarello V., Ginter S., Han L., Van Ness P., Allore H., Tinetti M. (2005). Modifiable risk factors for nursing home-acquired pneumonia. *Clinical Infectious Diseases*.

[17] Barnes C. M. (2014). Dental hygiene intervention to prevent nosocomial pneumonias. *Journal of Evidence Based Dental Practice*.

[18] Matsusaka K., Kawakami G., Kamekawa H. (2018). Pneumonia risks in bedridden patients receiving oral care and their screening tool: malnutrition and urinary tract infection-induced inflammation. *Geriatrics and Gerontology International*.

[19] Lopez A., Amaro R., Polverino E. (2012). Does health care associated pneumonia really exist?. *European Journal of Internal Medicine*.

[20] Bassim C. W., Gibson G., Ward T., Paphides B. M., DeNucci D. J. (2008). Modification of the risk of mortality from pneumonia with oral hygiene care. *Journal of the American Geriatrics Society*.

[21] Azarpazhooh A., Leake J. L. (2006). Systematic review of the association between respiratory diseases and oral health. *Journal of Periodontology*.

[22] Juthani-Mehta M., Van Ness P. H., McGloin J. (2014). A cluster-randomized controlled trial of a multicomponent intervention protocol for pneumonia prevention among nursing home elders. *Clinical Infectious Diseases*.

[23] Sjögren P., Wårdh I., Zimmerman M., Almståhl A., Wikström M. (2016). Oral care and mortality in older adults with pneumonia in hospitals or nursing homes: systematic review and meta-analysis. *Journal of the American Geriatrics Society*.

[24] Goll C. A., Swearingen P. L., Keen J. H. (2001). Mechanical ventilation. *Manual of Critical Care Nursing: Nursing Interventions and Collaborative Management*.

[25] Sole M. L., Byers J. F., Sole M. L., Lamborn M. L., Hartsborn J. (2001). Ventilatory assistance. *Introduction to Critical Care Nursing*.

[26] Deutsch J. M., Lynn McHale D. J., Carlson K. K. (2001). Endotracheal tube care. *AACN Procedure Manual for Critical Care*.

[27] Registered Nurses Association of Ontario (2008). *Oral Health: Nursing Assessment and Interventions*.

[28] O’Connor L., Boltz M., Capezuti E., Fulmer T., Zwicker D. (2012). Oral health care. *Evidence-Based Geriatric Nursing Protocols for Best Practice*.

[29] Henneman E., Ellstrom K., John St. R. (1998). *Airway Management*.

[30] Sole M. L., Byers J. F., Ludy J. E., Zhang Y., Banta C. M., Brummel K. (2003). A multisite survey of suctioning techniques and airway management practices. *American Journal of Critical Care*.

[31] Brinkley C., FurrL A., Carrico R. (2004). Survey of oral care practices in US intensive care units. *American Journal of Infection Control*.

[32] Grap M. J., Munro C. L., Ashtiani B., Bryant S. (2003). Oral care interventions in critical care: frequency and documentation. *American Journal of Critical Care*.

[33] Phillips C. D., Adepoju O., Stone N. (2012). Asymptomatic bacteriuria, antibiotic use, and suspected urinary tract infections in four nursing homes. *BMC Geriatrics*.

[34] Sekizawa K., Ujiie Y., Itabashi S., Sasaki H., Takishima T. (1990). Lack of cough reflex in aspiration pneumonia. *The Lancet*.

[35] Nomura Y., Morozumi T., Nakagawa T. (2017). Site-level progression of periodontal disease during a follow-up period. *PLoS One*.

[36] Terpenning M. S., Taylor G. W., Lopatin D. E., Kerr C. K., Dominguez B. L., Loesche W. J. (2001). Aspiration pneumonia: dental and oral risk factors in an older veteran population. *Journal of the American Geriatrics Society*.

[37] Langmore S. E., Terpenning M. S., Schork A. (1998). Predictors of aspiration pneumonia: how important is dysphagia?. *Dysphagia*.

[38] Groher M. E. (1987). Bolus management and aspiration pneumonia in patients with pseudobulbar dysphagia. *Dysphagia*.

[39] DePippo K. L., Holas M. A., Reding M. J., Mandel F. S., Lesser M. L. (1994). Dysphagia therapy following stroke: a controlled trial. *Neurology*.

[40] Sumi Y., Kagami H., Ohtsuka Y., Kakinoki Y., Haruguchi Y., Miyamoto H. (2003). High correlation between the bacterial species in denture plaque and pharyngeal microflora. *Gerodontology*.

[41] Adachi M., Ishihara K., Abe S., Okuda K., Ishikawa T. (2002). Effect of professional oral health care on the elderly living in nursing homes. *Oral Surgery, Oral Medicine, Oral Pathology, Oral Radiology, and Endodontology*.

[42] Abe S., Ishihara K., Okuda K. (2001). Prevalence of potential respiratory pathogens in the mouths of elderly patients and effects of professional oral care. *Archives of Gerontology and Geriatrics*.

[43] Adachi M., Ishihara K., Abe S., Okuda K. (2007). Professional oral health care by dental hygienists reduced respiratory infections in elderly persons requiring nursing care. *International Journal of Dental Hygiene*.

[44] Przybyłowska D., Rubinsztajn R., Chazan R. (2015). The prevalence of oral inflammation among denture wearing patients with chronic obstructive pulmonary disease. *Advances in Experimental Medicine and Biology*.

[45] Krause R., Halwachs B., Thallinger G. G. (2016). Characterisation of *Candida* within the mycobiome/microbiome of the lower respiratory tract of ICU patients. *PLoS One*.

