# Supplementary material for: Factors That Affect Oral Care Outcomes for Institutionalized Elderly
Source: Int J Dent. 2018 Dec 10;2018:2478408. doi: 10.1155/2018/2478408 (PMC6311881; doi:10.1155/2018/2478408)
Supplement: Supplementary Materials — S1: file model specification; S2: file cutoff point of oral levels of Candida for fever. S1 Table: characteristics of the subjects analyzed in this study at the start of oral care. S2 Table: effects of oral care independence on fever according to participation in the oral care program and number of functional teeth. S3 Table: effects of the interaction of dietary or meal factors with the oral care program and the number of functional teeth on the incidence of fever. S4 Table: effects of the interactions between Candida and oral care and Candida and the number of functional teeth on fever. Figure S1: the observational periods of the subjects who participated in this study. Figure S2: time series plots of the incidence of fever and hospitalization for pneumonia. Figure S3: distribution of the incidences of fever, hospitalization, and pneumonia-related hospitalization in patients who did and did not receive the oral care program. Figure S4: distribution of the incidence of fever and pneumonia-related hospitalization over the one-year follow-up period among the subjects who underwent the oral care program. Figure S5: probability of remaining fever-free among the subjects receiving the oral care program. [file 2478408.f1.pdf]

S1 Table Characteristics of the subjects analyzed in this study at the start of oral care

Lifestyle independence

	Subjects with follow-up data one year before and after oral care (N=27)		Subjects who underwent oral care (N=72)		Subjects with more than one year of follow-up(N=57)						P-value
					Start of oral care		After 6 months		After 12 months		
	n	%	n	%	n	%	n	%	n	%	
**Certification of the need for long-term care**											
1	0	0	1	1.4							0.003
2	3	11.1	5	6.9	5	9.4	4	7.5	1	1.9	
3	9	33.3	20	27.8	16	30.2	17	32.1	12	22.6	
4	11	40.7	26	36.1	22	41.5	24	45.3	27	54.0	
5	4	14.8	20	27.8	10	18.9	8	15.1	10	20.0	
**The degree of independence in everyday life**											
A	5	18.5	9	12.5	9	17.0	10	18.9	10	18.9	0.513
B	19	70.4	53	73.6	37	69.8	36	67.9	36	71.7	
C	2	7.4	6	8.3	3	5.7	4	7.5	3	5.7	
J	1	3.7	4	5.6	4	7.5	3	5.7	2	3.8	
**Dementia degree of autonomy**											
Independent	0	0.0	4	5.6	3	5.7	2	4.7	0	0	0.097
I	1	3.7	3	4.2	3	5.7	3	7.0	2	5.3	
II a	0	0.0	4	5.6	3	5.7	2	4.7	2	5.3	
II b	6	22.2	13	18.3	11	20.8	9	20.9	8	21.1	
III a	9	33.3	23	32.4	18	34.0	10	23.3	12	31.6	
III b	3	11.1	6	8.5	5	9.4	8	18.6	8	21.1	
IV	7	25.9	16	22.5	9	17.0	9	20.9	6	15.8	
M	1	3.7	2	2.8	1	1.9	0	0	0	0	

Meal and food factors

	Subjects with follow-up data from one year before and after oral care (N=27)		Subjects who underwent oral care (N=72)		Subjects with more than one year of follow-up (N=57)						P-value
					Start of oral care		After 6 months		After 12 months		
	n	%	n	%	n	%	n	%	n	%	
**Meal care**											
Independent	18	66.7	45	62.5	37	69.8	35	66.0	22	41.5	0.022
Needs care	4	14.8	9	12.5	7	13.2	7	13.2	9	17.0	
Dependent	5	18.5	18	25.0	9	17.0	10	18.9	21	39.6	
**Food prescription**											
Ordinary food	12	44.4	29	40.3	22	41.5	19	35.8	19	35.8	0.761
Sliced food	3	11.1	9	12.5	9	17.0	9	17.0	7	13.2	
Pureed food	12	44.4	32	44.4	21	39.6	23	43.4	22	41.5	
Liquid food	0	0	0	0	1	1.9	2	3.8	3	5.7	
**Arousal during the meal**											
+	24	88.9	59	81.9	50	94.3	50	94.3	45	84.9	0.074
-	3	11.1	13	18.1	3	5.7	2	3.8	6	11.3	

Oral function, oral health status and independence

	Subjects with follow-up data from one year before and after oral care (N=27)		Subjects who underwent oral care (N=72)		Subjects with more than one year of follow-up(N=57)						
					Start of oral care		After 6 months		After 12 months		P-value
	n	%	n	%	n	%	n	%	n	%	
**Swallowing difficulty**											
Residue -	44	81.5	52	72.2	40	75.5	48	90.6	45	84.9	0.999<
Residue +	8	14.8	8	11.1	6	11.3	2	3.8	2	3.8	
Unable to swallow	2	3.7	1	1.4	1	1.9	3	5.7	6	11.3	
**Dentures**											
-	28	51.9	35	48.6	23	43.4	22	41.5	21	39.6	0.197
+	26	48.1	37	51.4	30	56.6	30	56.6	30	56.6	
**Need care or attention for tooth brushing**											
Independent	18	33.3	27	37.5	24	45.3	20	37.7	15	28.3	<0.001
Needs care	14	25.9	16	22.2	12	22.6	10	18.9	7	13.2	
Dependent	22	40.7	29	40.3	17	32.1	22	41.5	29	54.7	
Candida											
<750 cfu	36	66.7	40	55.6	15	28.3	21	39.6			0.162
>=750 cfu	18	33.3	22	30.6	33	62.3	16	30.2			

Fifty-seven subjects had more than one year of follow-up data. Changes in parameters were analyzed by Friedman test.

S2 Table Effects of oral care independence on fever according to participation in the oral care program and number of functional teeth
(A) With or without dentures, (B) Assistance with tooth brushing, (C) Food test (swallowing function)

		Model S2 (A)				Model S2 (B)				Model S2 (C)				Model S2 (D)			
		Coefficient	95% CI		P-value	Coefficient	95% CI		P-value	Coefficient	95% CI		P-value	Coefficient	95% CI		P-value
			Lower	Upper			Lower	Upper			Lower	Upper			Lower	Upper	
**Intercept**		0.285	-0.211	0.780	0.254	0.689	0.161	1.217	0.012	0.205	-0.133	0.543	0.229	0.066	-0.197	0.33	0.615
**Oral care**	-	Reference				Reference				Reference				Reference			
	+	0.724	0.176	1.273	0.011	0.636	0.106	1.166	0.020	0.675	0.069	1.280	0.030	0.935	0.324	1.547	0.003
**Number of functional teeth**		0.030	-0.005	0.064	0.087	0.018	-0.009	0.044	0.185	0.035	<0.001	0.070	0.052	0.045	0.011	0.079	0.010
**Interaction with number of functional teeth**																	
**Oral care**	-	Reference				Reference				Reference				Reference			
	+	-0.054	-0.099	-0.009	0.020	-0.04	-0.076	-0.004	0.028	-0.050	-0.096	-0.004	0.034	-0.064	-0.114	-0.015	0.012
**Dentures**																	
	-	Reference															
	+	-0.237	-0.706	0.232	0.315												
**Needs assistance with tooth brushing**																	
Independent						Reference											
Needs attention						-0.622	-1.16	-0.084	0.024								
Dependent						-0.647	-1.158	-0.136	0.014								
**Swallowing ability**																	
Possible										Reference							
Impossible										-0.435	-0.816	-0.054	0.026				
**Meal support**																	
Independent														Reference			
Needs support														-0.312	-0.622	-0.002	0.048

With or without dentures was not statistically significant. Needs attention and dependent for tooth brushing were statistically significant. Their coefficients were negative. Inability to swallow was statistically significant.

The results indicate that the oral care program was effective for subjects who were not fully independent during tooth brushing and for those with dysphagia. The effect of the oral care program was not associated with denture use. This may be because dedicated attention was paid to denture wearers and because detailed denture care instructions were provided for the nurses and caregivers.

S3 Table Effects of the interaction of dietary or meal factors with the oral care program and the number of functional teeth on the incidence of fever

Model S3 (A)

		Coefficient	95% CI		P-value
			Lower	Upper	
**Intercept**		0.222	-0.002	0.446	0.052
**Interaction with oral care and meal care**					
**Oral care (+)**	**Independent**	Reference			
	**Needs attention**	2.028	0.5	3.556	0.010
	**Needs assistance**	-0.222	-0.446	0.002	0.052
**Oral care (-)**	**Independent**	0.167	-0.205	0.538	0.371
	**Needs attention**	1.778	0.335	3.220	0.017
	**Needs assistance**	0.178	-0.435	0.790	0.562

Model S3 (B)

		Coefficient	95% CI		P-value
			Lower	Upper	
**Intercept**		0.417	0.042	0.791	0.030
**Interaction with oral care and food prescription**					
**Oral care (+)**	Ordinary	Reference			
	Sliced	1.250	-0.295	2.795	0.110
	Pureed	-0.167	-0.641	0.307	0.483
**Oral care (-)**	Ordinary	-0.083	-0.586	0.419	0.740
	Sliced	0.917	-0.475	2.308	0.192
	**Pureed**	0.333	-0.294	0.960	0.290

Model S3 (C)

		Coefficient	95% CI		P-value
			Lower	Upper	
**Intercept**		0.500	0.210	0.790	0.001
**Interaction with oral care and food prescription**					
**Oral care (+)**	**Arousal(+)**	Reference			
	**Arousal(-)**	-0.167	-0.896	0.563	0.648
**Oral care (-)**	**Arousal(+)**	-0.167	-0.541	0.208	0.375
	**Arousal(-)**	2.500	0.471	4.529	0.017

Model S3 (D)				
	Coefficient	95% CI		P-value
		Lower	Upper	
**Intercept**	1.193	-4392.762	4395.148	0.999<
**Interaction with number of functional teeth and meal care**				
**Independent**	-0.017	-411.164	411.13	0.999<
**Needs attention**	0.033	-507.696	507.763	0.999<
**Needs assistance**	-0.056	-587.035	586.923	0.999<

Model S3 (E)				
	Coefficient	95% CI		P-value
		Lower	Upper	
**Intercept**	<0.001	<0.001	<0.001	0.999<
**Interaction with number of functional teeth and food prescription**				
**Ordinary**	0.033	0.011	0.055	0.004
**Sliced**	0.136	0.045	0.228	0.004
**Pureed**	<0.001	<0.001	<0.001	0.999<

Model S3 (F)				
	Coefficient	95% CI		P-value
		Lower	Upper	
**Intercept**	-0.102	-0.373	0.169	0.451
**Interaction with number of functional teeth and arousal at meal time**				
**Arousal(+)**	0.024	-0.002	0.049	0.067
**Arousal(-)**	0.047	-0.017	0.111	0.148

The interactions between the need for attention at meal time and participation/nonparticipation in the oral care program and between nonarousal during the meal and no oral care program had statistically significant effects on the incidence of fever. The interactions between dietary prescription and oral care program were not statistically significant. In contrast, the interaction between the number of functional teeth and ordinary and sliced food prescriptions had significant effects on the risk of fever. The coefficient for the prescription of sliced food was higher for the prescription of ordinary food.

S4 Table Effect of the interactions of *Candida* and oral care and candida and number of functional teeth on fever

Model S4 (A)

		Coefficient	95% CI Lower	95% CI Upper	P-value
**Intercept**		0.389	0.094	0.684	0.011
**Interaction with oral care and oral levels of *Candida***					
**Oral care (－)**	***Candida* <750 cfu**	Reference			
	***Candida* >=750 cfu**	0.278	-0.344	0.899	0.373
**Oral care (＋)**	***Candida* <750 cfu**	-0.167	-0.537	0.203	0.370
	***Candida* >=750 cfu**	1.056	0.198	1.913	0.017

Model S4 (B)

		Coefficient	95% CI Lower	95% CI Upper	P-value
**Intercept**		0.389	0.094	0.684	0.011
**Interaction with number of functional teeth and oral levels of *Candida***					
Candida	**<750 cfu**	0.030	0.002	0.059	0.038
	**>=750 cfu**	0.071	0.028	0.114	0.002

Coefficient of interaction of oral *Candia*>=750 cfu and oral care (+) was statistically significant and its coefficient was positive. The results indicated that the risk of pyrexia may increase by the oral care for the subjects with higher levels of *Candida*. And interactions of *Candida* and number of functional teeth had statistically significant coefficient. Coefficient of Candida levels >=750 cfu was higher than that of <750 cfu.

S1 File Model Specification

Model 1

Multilevel random slope and random intercept model for the incidence of fever.

Below, fixed effects (random effects or error terms) are denoted by Greek letters (alphabet).

Model 1 Subjects and oral care are indexed by *ij*.

$$P_o(X_o) = \pi_{0i} + \pi_{1i}(\text{Number of functional teeth})_i + \varepsilon_{1ij} \sum_{m=1}^{2} \pi_{2i}^{(m)}(\text{Oral care})_i + \sum_{m=1}^{2} \pi_{2i}^{(m)}(\text{Oral care})_{ij} \times (\text{Number of functional teeth})_i + \varepsilon_{2ij}$$

$$where\ e_i \sim N(0, \delta_e^2)\ and\ e_i \sim N(0, \delta_e^2)$$

Data structure: Subjects, oral care

Random effects

Oral care level: Intercept, random slope for oral care

Random effect covariance: variance component

Where *Xo*: Fever, X_1: Hospitalization, X_2: Hospitalization for pneumonia for Model 1(A),(B) and (C), respectively

SPSS Syntax

```
*Generalized Linear Mixed Models.

GENLINMIXED

  /DATA_STRUCTURE SUBJECTS=Subjects

  /FIELDS TARGET=X0 TRIALS=NONE OFFSET=NONE

  /TARGET_OPTIONS DISTRIBUTION=POISSON LINK=IDENTITY

  /FIXED EFFECTS=Oralcare Numberoffunctionalteeth Oralcare *Numberoffunctionalteeth USE_INTERCEPT=TRUE

  /RANDOM EFFECTS=Oralcare USE_INTERCEPT=TRUE SUBJECTS=Subjects COVARIANCE_TYPE=VARIANCE_COMPONENTS

  /BUILD_OPTIONS TARGET_CATEGORY_ORDER=ASCENDING INPUTS_CATEGORY_ORDER=ASCENDING MAX_ITERATIONS=100

    CONFIDENCE_LEVEL=95 DF_METHOD=RESIDUAL COVB=MODEL PCONVERGE=0.000001(ABSOLUTE) SCORING=0

    SINGULAR=0.000000000001

  /EMMEANS_OPTIONS SCALE=ORIGINAL PADJUST=LSD.
```

Models 2, 3

Multilevel random slope and random intercept model for the incidence of fever according to dietary or meal factors and *Candida.*

Below, fixed effects (random effects or error terms) are denoted by Greek letters (alphabet).

Model 1 Subjects and oral care are indexed by *ij*.

$$P_o(\text{Fever}) = \pi_{0i} + \pi_{1i}(\text{Number of functional teeth})_i + \varepsilon_{1ij} \sum_{m=1}^{2} \pi_{2i}^{(m)}(\text{Oral care})_i + \sum_{m=1}^{2} \pi_{2i}^{(m)}(\text{Oral care})_{ij} \times (\text{Number of functional teeth})_i + \sum_{m=1}^{2} \pi_{2i}^{(m)}\,(X_o)_i + \varepsilon_{2ij}$$

$$where\ e_i \sim N(0, \delta_e^2)\ and\ e_i \sim N(0, \delta_e^2)$$

Data structure: Subjects, Oral care

Random effects

Oral care level: Intercept, random slope for oral care

Random effect covariance: Variance component

Where *Xo*: Meal care, X_1: Food prescription, X_2: Arousal during meals, and X_4: Oral levels of Candida for Model 2(A), (B), (C) and (D), respectively

SPSS Syntax

```
*Generalized Linear Mixed Models.
GENLINMIXED
  /DATA_STRUCTURE SUBJECTS=Subjects
  /FIELDS TARGET=Pyrexia TRIALS=NONE OFFSET=NONE
  /TARGET_OPTIONS DISTRIBUTION=POISSON LINK=IDENTITY
  /FIXED EFFECTS=Oralcare Numberoffunctionalteeth Oralcare *Numberoffunctionalteeth Xo USE_INTERCEPT=TRUE
  /RANDOM EFFECTS=Oralcare USE_INTERCEPT=TRUE SUBJECTS=Subjects COVARIANCE_TYPE=VARIANCE_COMPONENTS
  /BUILD_OPTIONS TARGET_CATEGORY_ORDER=ASCENDING INPUTS_CATEGORY_ORDER=ASCENDING MAX_ITERATIONS=100
    CONFIDENCE_LEVEL=95 DF_METHOD=RESIDUAL COVB=MODEL PCONVERGE=0.000001(ABSOLUTE) SCORING=0
    SINGULAR=0.000000000001
  /EMMEANS_OPTIONS SCALE=ORIGINAL PADJUST=LSD.
```

Model 3

Generalized linear model for the incidence of fever according to the number of functional teeth, dietary or meal factors and *Candida.*

Below, error terms are denoted by Greek letters (alphabet).

Subjects are indexed by i.

$$P_o(\text{Fever}) = \alpha_1(\text{Number of functional teeth})_i + \alpha_2 \sum_{m=1}^{n} (\text{Meal care})_i$$

$$+ \alpha_3 \sum_{m=1}^{n} (\text{Food prescripton})_i + \alpha_4 \sum_{m=1}^{n} (\text{Apousal})_i + \alpha_5 \sum_{m=1}^{n} (Candida)_i + \varepsilon_i$$

SPSS Syntax

```
GENLIN Pyrexia BY Mealcare Foodprescription Arousal Candida (ORDER=ASCENDING) WITH Functionalteeh
  /MODEL Mealcare Foodprescription Arousal Candida Functionalteeh INTERCEPT=YES
 DISTRIBUTION=POISSON LINK=LOG
  /CRITERIA METHOD=FISHER(1) SCALE=1 COVB=MODEL MAXITERATIONS=100 MAXSTEPHALVING=5
    PCONVERGE=1E-006(ABSOLUTE) SINGULAR=1E-012 ANALYSISTYPE=3(WALD) CILEVEL=95 CITYPE=WALD
    LIKELIHOOD=FULL
  /MISSING CLASSMISSING=EXCLUDE
  /PRINT CPS DESCRIPTIVES MODELINFO FIT SUMMARY SOLUTION.
```

Model 4

Cox's proportional hazard model for the time between the start of the oral care program and the incidence of fever according to the number of functional teeth, dietary and meal factors and *Candida.*

$$h(t|x) = h_0(t)\exp(a_1x_1 + a_2x_2 + a_3x_3 + a_4x_4 + a_5x_5)$$

SPSS Syntax

```
BOOTSTRAP
  /SAMPLING METHOD=SIMPLE
  /VARIABLES INPUT=O1 OT1 v12 vc06 v26 v07 v08
  /CRITERIA CILEVEL=95 CITYPE=PERCENTILE NSAMPLES=1000
  /MISSING USERMISSING=EXCLUDE.
COXREG OT1
  /STATUS=O1(1)
  /CONTRAST (v12)=Indicator
  /CONTRAST (v26)=Indicator
  /CONTRAST (v07)=Indicator
  /CONTRAST (v08)=Indicator
  /METHOD=ENTER vc06 v07 v08 v12 v26
  /PRINT=CI(95)
  /CRITERIA=PIN(.05) POUT(.10) ITERATE(20).
```

S2 File

Colony counts of *Candida* depend on the number of samples. With swab sampling, it is impossible to obtain a uniform number of samples. Therefore, we dichotomized the colony count of *Candida* using the following methodology.

(A) ROC curves were used to set the cutoff point of oral levels of *Candida* for fever.

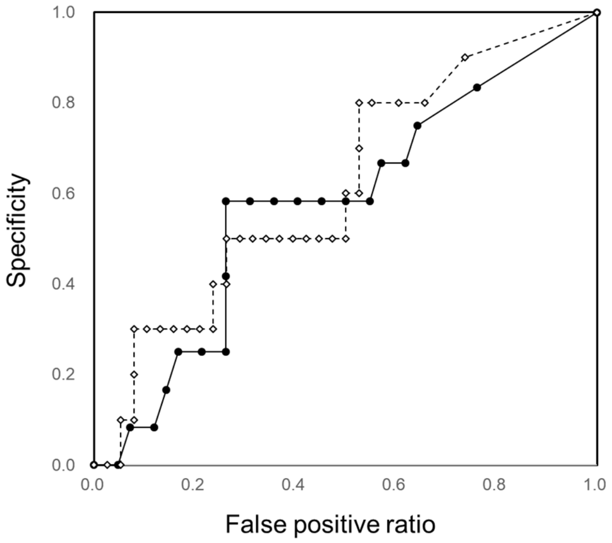

The solid line indicates the 27 subjects who did and did not receive the oral care intervention. The dotted line indicates the data for 53 subjects for one year after receiving oral care. The optimal cut off points were between 700 cfu and 785 cfu for the 27 subjects who did and did not receive oral care and between 735 cfu and 785 cfu for the 53 subjects with data for one year after receiving oral care. Therefore, in this study, the cut-off point was conventionally set as 750 cfu.

(B) Cross tabulations of oral levels of Candida and fever

Data			*Candida*	
			<750 cfu	750 cfu<=
With or without oral care	Fever	+	5	7
		-	31	11
Oral care	Fever	+	5	5
		-	10	28

(C) Precision of the cut-off point

Data	Sensitivity	Specificity	Positive predictive vale	Negative predictive vale	AUR	Likelihood ratio
With or without oral care	0.58	0.74	0.39	0.86	0.58	1.77
Oral care	0.50	0.74	0.33	0.85	0.62	1.47

AUR: Area under the ROC curve

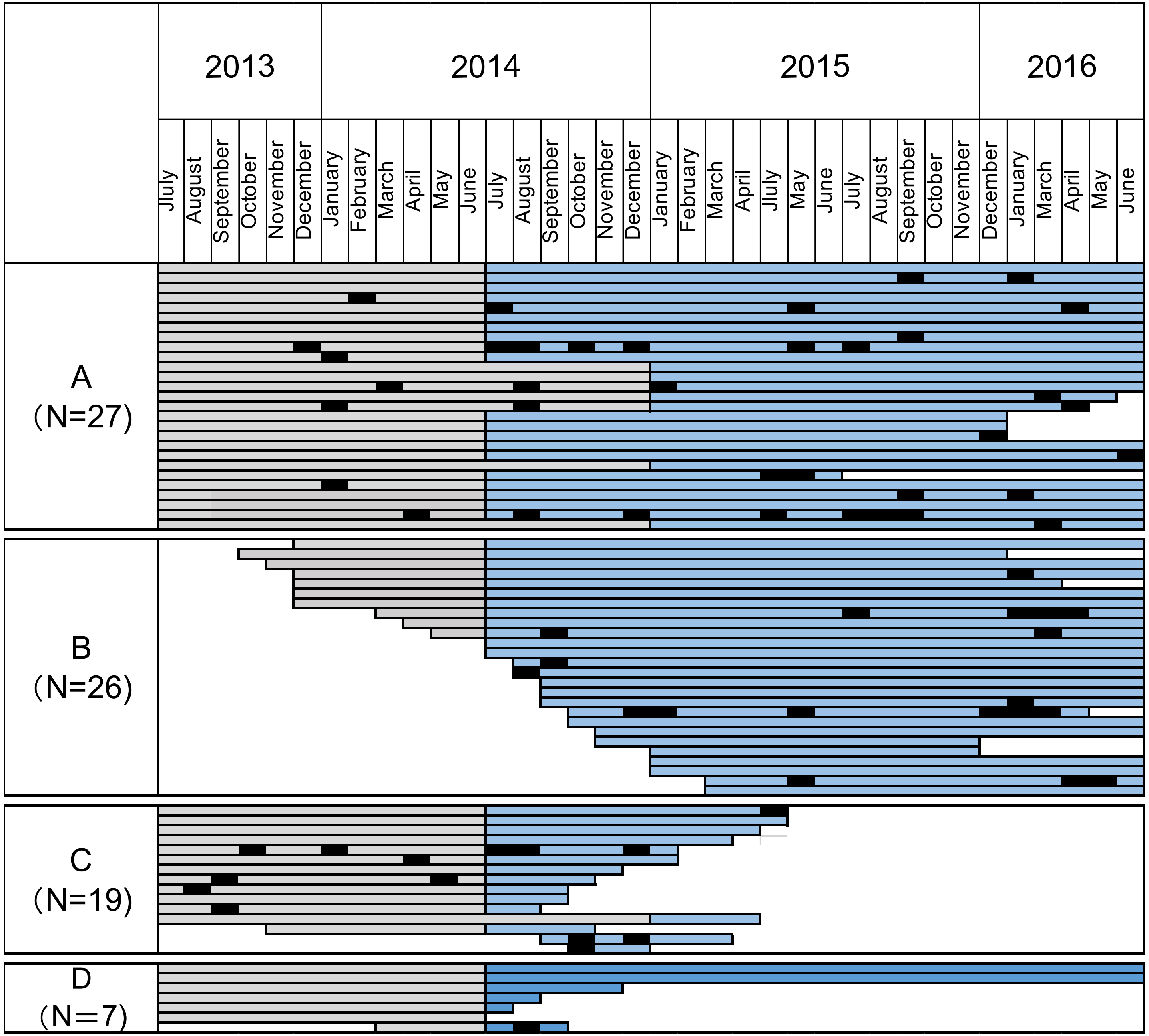
2013
2014
2015
2016
July
August
September
October
November
December
January
February
March
April
May
June
July
August
September
October
November
December
January
February
March
April
Jluly
May
June
July
August
September
October
November
December
January
March
April
May
June
A
(N=27)
B
(N=26)
C
(N=19)
D
(N=7)

S1 Figure

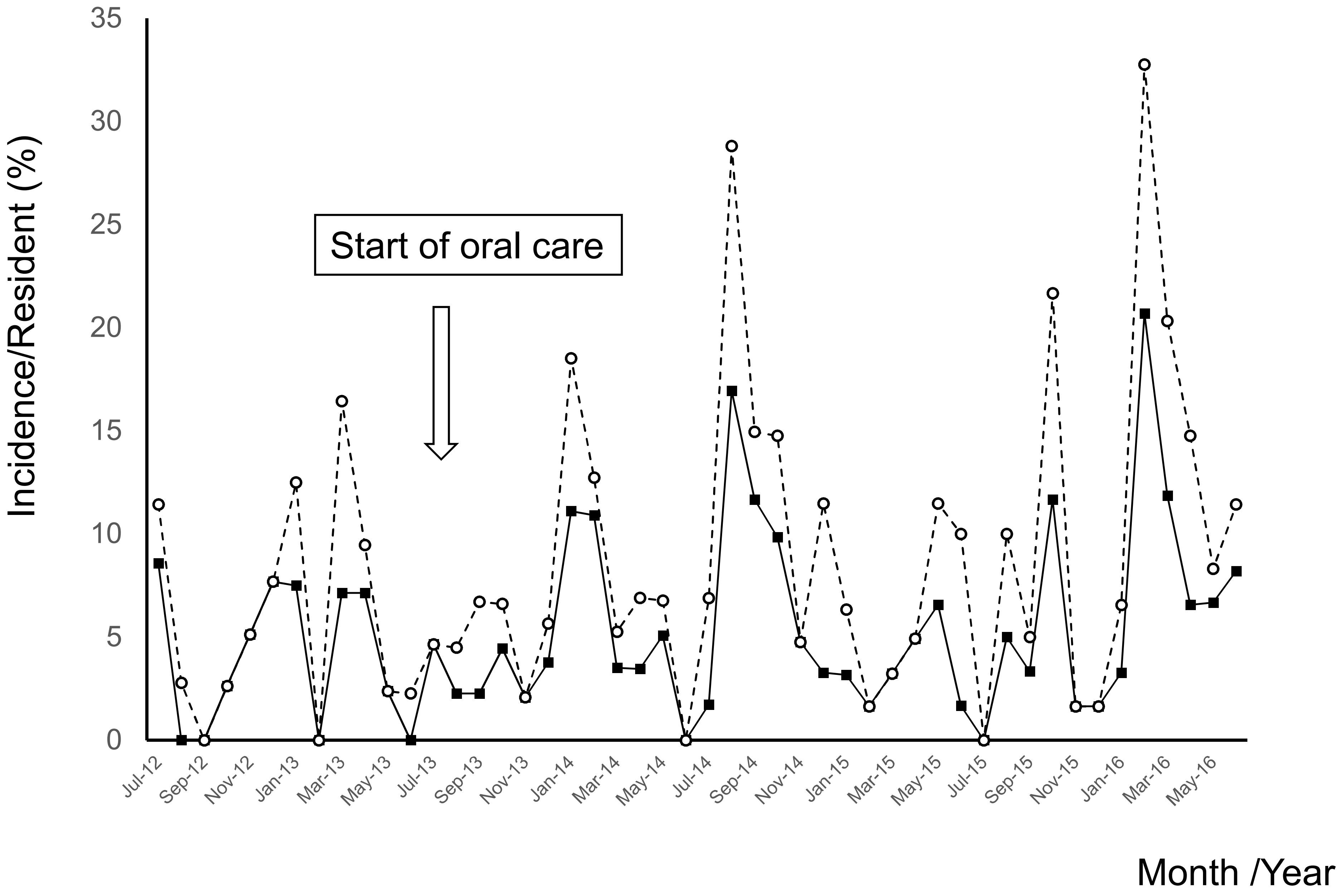
35
30
25
20
15
10
5
0
Incidence/Resident (%)
Start of oral care
Jul-12
Sep-12
Nov-12
Jan-13
Mar-13
May-13
Jul-13
Sep-13
Nov-13
Jan-14
Mar-14
May-14
Jul-14
Sep-14
Nov-14
Jan-15
Mar-15
May-15
Jul-15
Sep-15
Nov-15
Jan-16
Mar-16
May-16
Month /Year

S2 Figure

Distribution of the incidence of fever, hospitalization and hospitalization for pneumonia with or without oral care intervention

(A)

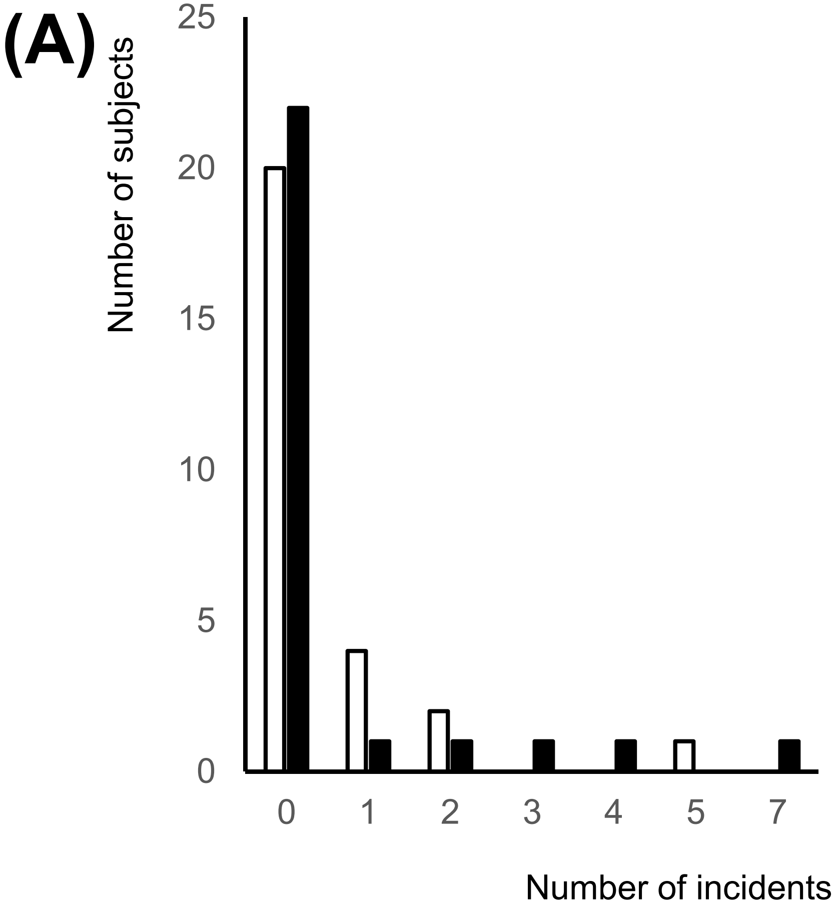

Fever

(B)

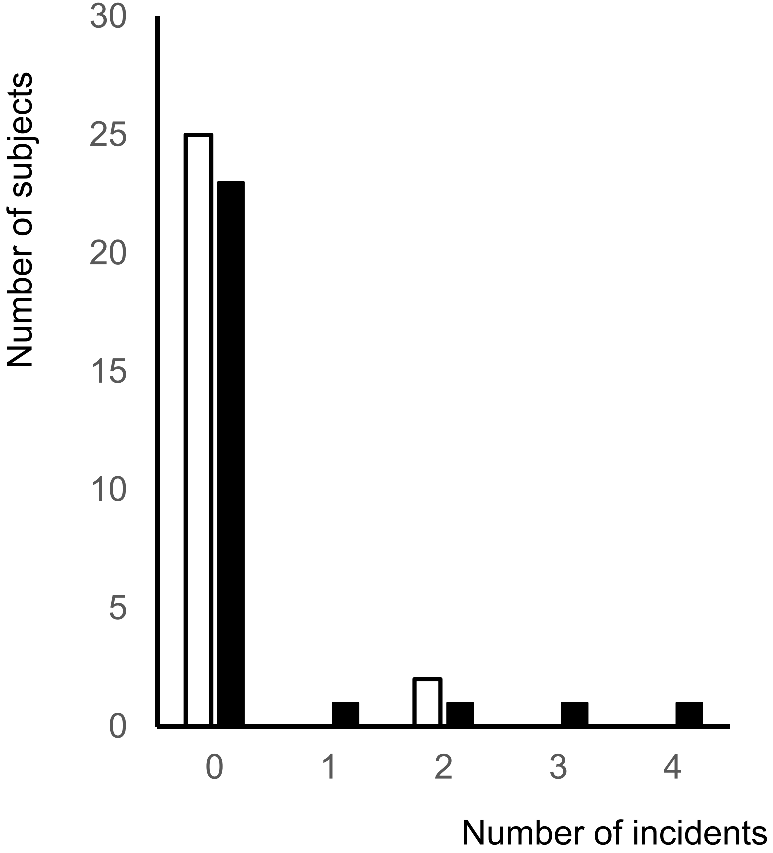

Hospitalization for pneumonia

S3 Figure

Distribution of incidence of fever, hospitalization and hospitalization for pneumonia during the 1-year follow-up period

(A)

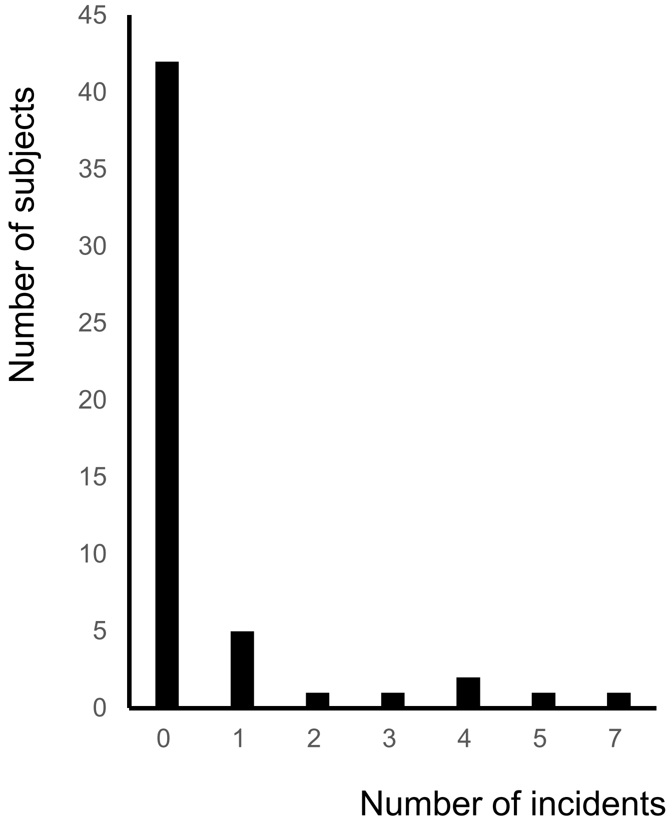

Fever

(B)

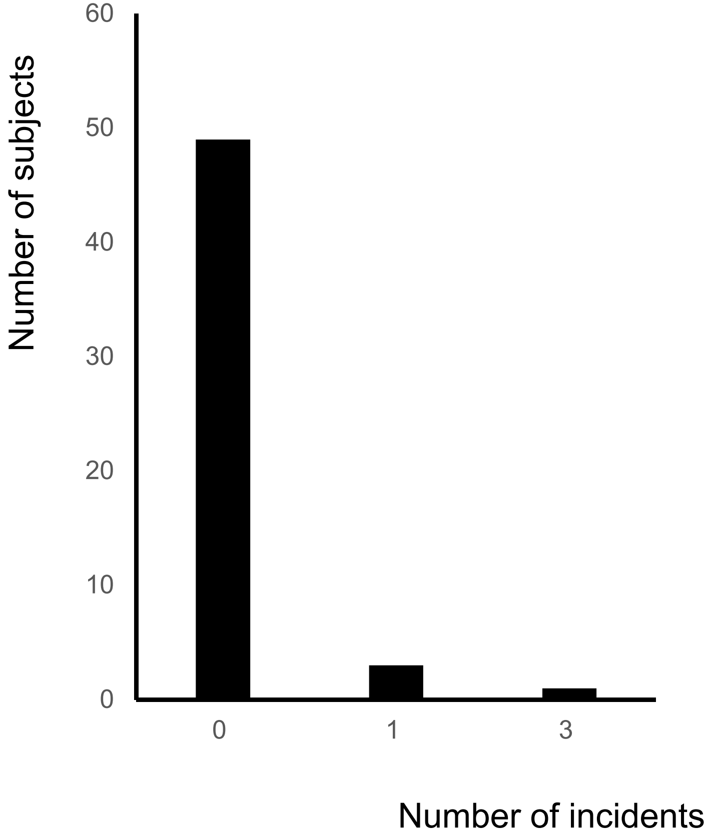

Hospitalization for pneumonia

S4 Figure

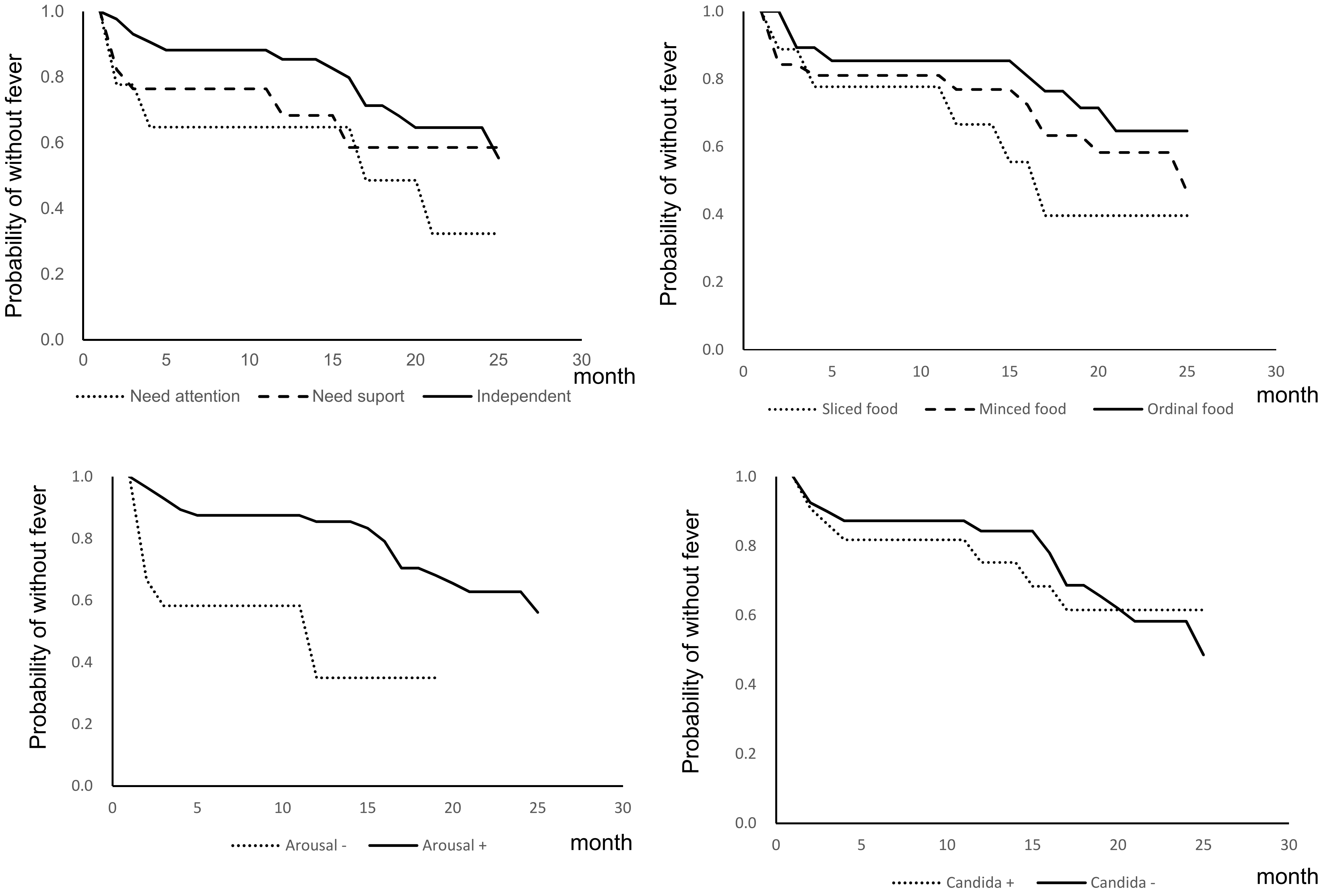
1.0
0.8
0.6
0.4
0.2
0.0
Probability of without fever
0
5
10
15
20
25
30
month
Need attention
Need suport
Independent
1.0
0.8
0.6
0.4
0.2
0.0
Probability of without fever
0
5
10
15
20
25
30
month
Sliced food
Minced food
Ordinal food
1.0
0.8
0.6
0.4
0.2
0.0
Probability of without fever
0
5
10
15
20
25
30
Arousal -
Arousal +
month
1.0
0.8
0.6
0.4
0.2
0.0
Probability of without fever
0
5
10
15
20
25
30
month
Candida +
Candida -

S5 Figure
